# Tetrodotoxin prevents heat-shock induced granule cell dispersion in hippocampal slice cultures

**DOI:** 10.3389/fcell.2022.906262

**Published:** 2022-08-25

**Authors:** Ala Ahrari, Maurice Meseke, Eckart Förster

**Affiliations:** Department of Neuroanatomy and Molecular Brain Research, Institute of Anatomy, Ruhr- Universität Bochum, Bochum, Germany

**Keywords:** dentate gyrus, reelin, cajal-retzius cells, febrile seizure (FS), epilepsy, extracellular matrix

## Abstract

Granule cell dispersion (GCD) has been associated as a pathological feature of temporal lobe epilepsy (TLE). Early-life epileptiform activity such as febrile seizures has been proposed to have a causal link to developing chronic TLE. During postnatal development, the hippocampus may be particularly vulnerable to hyperexcitability-induced insults since neuronal migration and differentiation are still ongoing in the hippocampus. Further, the extracellular matrix (ECM), here in particular the protein reelin, has been implicated in the pathophysiology of GCD. Thus, loss of reelin-expressing cells, Cajal-Retzius cells and subsets of interneurons, may be related to GCD. To study the possible role of febrile seizures, we previously induced GCD *in vitro* by subjecting hippocampal slice cultures to a transient heat-shock, which was not accompanied by loss of Cajal-Retzius cells. In order to examine the mechanisms involved in heat-shock induced GCD, the present study aimed to determine whether such dispersion could be prevented by blocking cellular electrical activity. Here we show that the extent of heat-shock induced GCD could be significantly reduced by treatment with the sodium channel blocker tetrodotoxin (TTX), suggesting that electrical activity is an important factor involved in heat-shock induced GCD.

## Introduction

GCD has been implicated in pathological conditions of human temporal lobe epilepsy (TLE) and hippocampal sclerosis (Houser, 1990; Armstrong, 1993; Blümcke et al., 2009). GCD has also been studied in animal models of epilepsy (Suzuki et al., 1995; Riban et al., 2002), where it was observed upon kainic acid (KA) intrahippocampal injections, with morphological similarity to granule cell malpositioning in the reeler mutant mouse lacking reelin expression. The protein reelin has been shown to be important for proper lamination of dentate granule cell layer (GCL) (Gong et al., 2007; Duveau et al., 2010) while changes in reelin expression have been implicated in epilepsy (Dazzo et al., 2015) and neuropsychiatric disorders (Folsom and Fatemi, 2013). Further, chronically infusing recombinant reelin into the hippocampus reduced GCD (Müller et al., 2009). *In vitro* experiments on organotypic hippocampal slice cultures (OHSCs) (Orcinha et al., 2016) pointed to loss of reelin-expressing interneurons in the hilus as the main cause for KA-induced GCD. In contrast, in interneuron-specific reelin knockout mice (Pahle et al., 2019), no GCD was observed, implicating that the reelin-expressing interneuron cell populations may not be essential for granule cell lamination. In line with neuronal migration defects related to reelin deficiency, decreased reelin expression was observed in tissue dissected from patients with TLE (Haas et al., 2002), and in the KA animal model of epilepsy (Heinrich, 2006).

Studies on animal models and children with febrile seizures suggest a causal relationship between febrile seizures and the propensity to develop TLE later in life (French et al., 1993; Thom et al., 2002; Patterson et al., 2014). The role of febrile seizures and their tendency to exacerbate epileptic events in humans, have been experimentally addressed in animal models (Liu et al., 1993; Dube et al., 2000). Thus, rats were subjected to hyperthermia-induced seizures and then injected with KA to induce epilepsy (Dube et al., 2000). In consequence, 100% of the animals in the hyperthermia group developed hippocampal seizures, but without hyperthermia only 25% developed seizures. Similarly, when neonate rats were exposed to prolonged hyperthermic seizures, 35% displayed spontaneous seizures as adult animals (Dubé et al., 2006). Although 30% of TLE patients with GCD could be linked to febrile seizures in early childhood (Lurton et al., 1998), it is to be noted that GCD has also been observed in normal, non-epileptic paediatric patients (Roy et al., 2020).

To examine the potential role of fever on the formation of GCD, we established a model wherein OHSCs were subjected to increased temperature (Weninger et al., 2021). In this model, we found that transiently elevated temperature alone caused GCD, and that this phenomenon was not accompanied by changes in the number of Cajal-Retzius cells. As there is evidence for a relationship between electrical activity and GCD in various epilepsy models, the present study aimed to determine whether GCD observed in the heat-shock model could be reduced by blocking electrical activity with the sodium channel blocker tetrodotoxin (TTX).

## Results

### Tetrodotoxin prevents heat-shock induced granule cell dispersion

**FIGURE 1:**
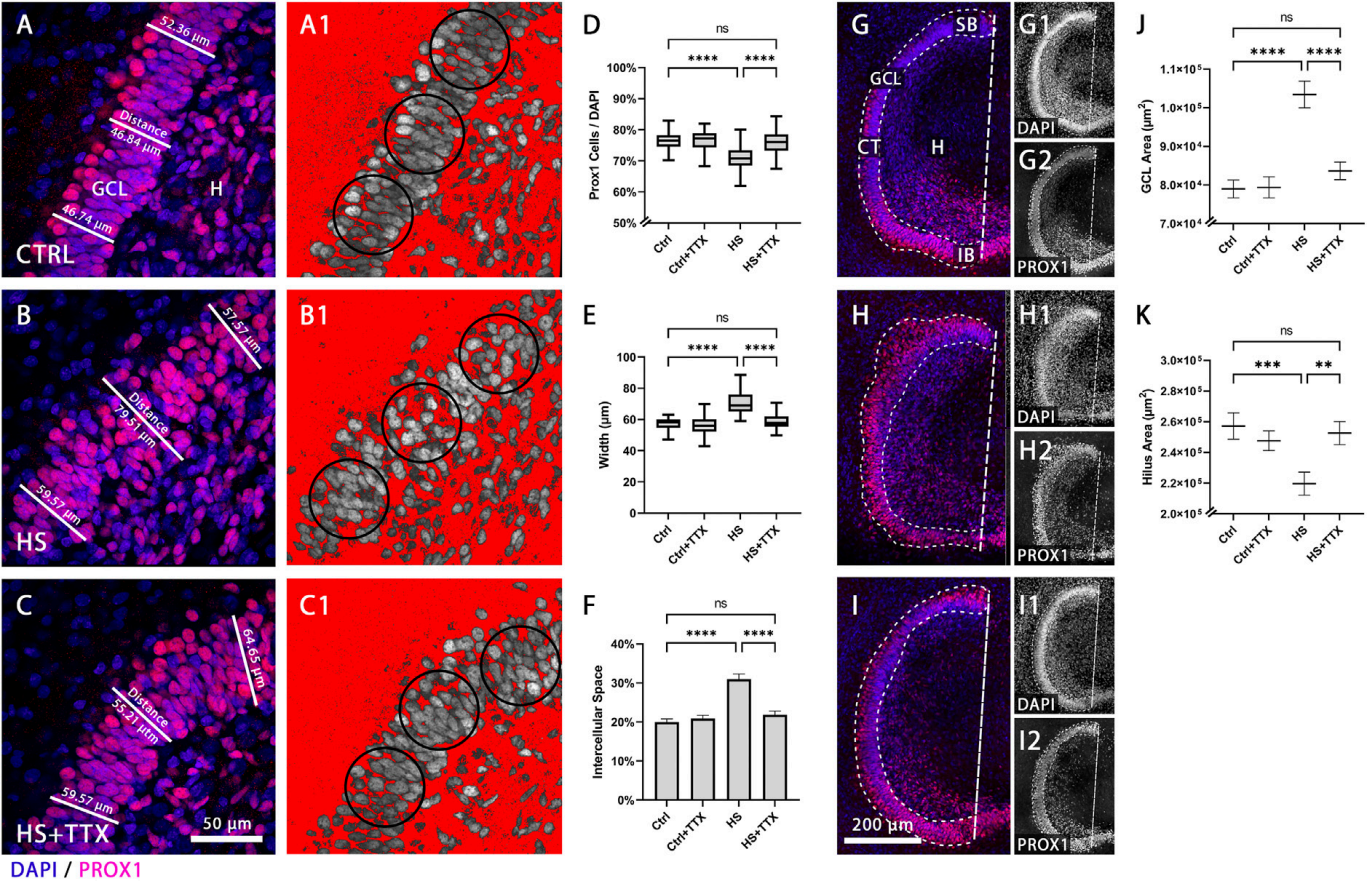
Granule cell density and dentate gyrus size in TTX-treated heat-shocked hippocampal slice cultures. Representative 60 × magnification images of animal- and section-matched OHSCs (*n* = 16 animals, 46 OHSCs per condition) demonstrating the compactness of the granule cell layer (GCL), as shown with immunofluorescent staining of Prox1-positive granule cells (red) counterstained with DAPI (blue) in the GCL **(A–C)**. **(A)** Control (CTRL) condition exhibited a sharp well-defined GCL, which was reduced after **(B)** heat-shock (HS). Following **(C)** TTX treatment during heat-shock, the GCL was more compact, comparable to control. The packaging density of granule cells within the GCL was assessed with an inverted threshold mask (**A1-C1**; shown in red), set to visualise the distance between Prox1-positive nuclei in the GCL. In comparison to **(A1)** control, after **(B1)** heat-shock there was an increase in space between the Prox1-positive granule cells nuclei, which could be reduced with **(C1)** TTX treatment. **(D)** The average percentage of Prox1-positive granule cells normalised to the whole cell number (DAPI-positive nuclei) in the region of interest (circular masks of 2,000 μm^2^) revealed a significant reduction in granule cell density in the dentate gyrus (*p* < 0.0001; ANOVA; Boxplot with median, 25% and 75% quartile) following heat-shock, and this effect was attenuated upon treatment with TTX, whereas TTX treatment of control showed no effect. **(E)** Measuring the average width of the GCL demonstrated a significant increase in width after heat-shock (*p* < 0.0001; Friedman; Boxplot with median, 25% and 75% quartile) which was found to be reduced in TTX-treated heat-shock slices, and therefore more comparable to control. No significant change was seen by treatment of control slices with TTX. **(F)** Similarly, the percentage of space between granule cell nuclei within the GCL was significantly increased following heat-shock (*p* < 0.0001; ANOVA; mean ± SEM), but not in the TTX-treated control or in TTX-treated heat-shock slice cultures. Representative 20 × magnification images for size measurement of GCL and hilus in control and following TTX treatment are shown in **(G–I)** (*n* = 16 animals, 46 OHSCs per condition). Images demonstrate **(G)** control (CTRL) with its sharp GCL border, and dispersion of the sharp border after **(H)** heat-shock (HS), and such dispersion was not observed with **(I)** TTX treatment during heat-shock. The whole GCL area was determined by drawing a mask around the GCL, stained with DAPI and for Prox1. The hilus area was determined (see methods) as the area outlined by the GCL and the dashed straight line from the suprapyramidal blade to the infrapyramidal blade (dashed line; **G,I**). Splitting the overlay into single channels for Prox1 **(G1, H1, I1)** and DAPI **(G2, H2, I2)** staining emphasises dispersion of Prox1-positive cells of the suprapyramidal blade after heat-shock **(H2)** compared to control **(G2)**, which is rescued following TTX treatment combined with heat-shock **(I2)**. **(J)** The area of the GCL significantly increased due to heat-shock, but was preserved after TTX treatment. The area of control slices treated with TTX remained unchanged (ANOVA; Tukey’s post test; mean ± SEM). **(K)** The hilus area was significantly reduced in heat-shocked slice cultures, but preserved following TTX treatment, again resembling control and TTX-treated control slices (ANOVA; Tukey’s post test; mean ± SEM). CTRL: control; GCL: granule cell layer; **(H)** hilus; HS: heat-shock; SB: suprapyramidal blade; CT: crest; IB: infrapyramidal blade; TTX: tetrodotoxin; *p < 0.05, **p < 0.01, ***p < 0.001, and ****p < 0.0001).

The density of Prox1-positive cells in the GCL was analysed (*n* = 16 animals, 46 OHSCs per condition) to determine the effect of the sodium-channel blocker TTX (1 µM) on GCL compactness. Following heat-shock, GCL compactness was reduced in comparison to control (Figures 1A,B). In contrast, granule cells in TTX-treated heat-shock OHSCs were found to be more compact, i.e., similar to control (Figure 1C). There was a significant reduction (*p* < 0.0001; ANOVA; Figure 1D) in granule cell density (Prox1-positive nuclei normalised to DAPI-positive nuclei) across the dentate gyrus (6.2% ± 0.8%; *p* < 0.0001; Tukey’s post test; Figure 1D) following heat-shock. TTX treatment alone showed no significant impact on GCL morphology (0.7% ± 0.8%; *p* = 0.86; Tukey’s post test; Figure 1D), whereas treatment with TTX reduced heat-shock induced GCD, comparable to control (0.6% ± 0.8%; *p* = 0.88; Tukey’s post test; Figure 1D).

To further analyse the effect of TTX on heat-shock induced GCD, the GCL width was measured (Figures 1A–C). In TTX-treated heat-shock slices cultures the GCL was found to be more compact when compared to heat-shock treatment alone (Figures 1B,C), with fewer Prox1-positive cells detached from the compact GCL. Statistical analysis (*p* < 0.0001; Friedman; Figure 1E) revealed a significant increase in GCL width following heat-shock (increase of 18.6%; *p* < 0.0001; Dunn’s post test; Figure 1E). Treatment with TTX alone caused no significant difference in width (decrease of 3.6%; *p* = 0.99; Dunn’s post test; Figure 1E), and the GCL width was comparable to control upon TTX treatment in heat-shock (decrease of 0.8%; *p* = 0.99; Dunn’s post test; Figure 1E).

Moreover, there was a significant increase of 10.98% ± 1.36% space between Prox1-positive nuclei in heat-shock OHSCs compared to control (*p* < 0.0001; ANOVA; Figure 1F), demonstrated in Figures 1A1–C1 *via* an inverted threshold mask (red) that selected the area surrounding Prox1-positive nuclei. The compactness observed in control (Figure 1A1) was lost following heat-shock (Figure 1B1). Treatment with TTX alone caused no significant change in the space between Prox1-positive nuclei (0.92% ± 1.36% space; *p* = 0.91; Tukey’s post test; Figure 1F), and GCL compactness upon TTX treatment during heat-shock was similar to control (1.90% ± 1.36% space; *p* = 0.51; Figures 1F, C1) in contrast when compared to heat-shock treatment alone (Figures 1F, B1).

Furthermore, the hilus area was measured following TTX treatment. In control, there was a clear border between GCL and hilus, (Figure 1G; single channel representation for DAPI in G1 and Prox1 staining in G2), which was lost following heat-shock (Figure 1H; H1-DAPI; H2-Prox1). Treatment with TTX (Figure 1I; I1-DAPI; I2-Prox1) reduced such dispersion. The total area of the GCL itself was measured to account for dispersion into the molecular layer and the hilus. There was a 23.6% increase in GCL area following heat-shock (mean difference: 24,459 μm^2^; *p* < 0.0001; Tukey’s post test; Figure 1J). The GCL area of OHSCs did not significantly change with TTX treatment in control (0.5% GCL area increase after TTX; mean difference: 412 μm^2^; *p* = 0.99; Tukey’s post test; Figure 1J) and following TTX treatment in heat-shock (5.6% GCL area increase after TTX; mean difference: 4,673 μm^2^; *p* = 0.99; Tukey’s post test; Figure 1J). Together, the analyses on the GCL revealed a reduction of heat-shock-induced GCD upon TTX treatment.

On the contrary, analysis of OHSCs (*p* < 0.0001; ANOVA; Figure 1K) exhibited a significant decrease by 17.1% in the hilus area of heat-shocked OHSCs (mean difference: 37,565 μm^2^, *p* = 0.0005; Tukey’s post test; Figure 1K) compared to control. In TTX-treated heat-shock OHSCs the hilus area did not significantly differ from control (1.8% hilus area decrease; mean difference 4,590 μm^2^; *p* = 0.96; Tukey’s post test), which was also true for TTX-treated control (3.8% hilus area decrease; mean difference 9,580 μm^2^; *p* = 0.73).

**FIGURE 2:**
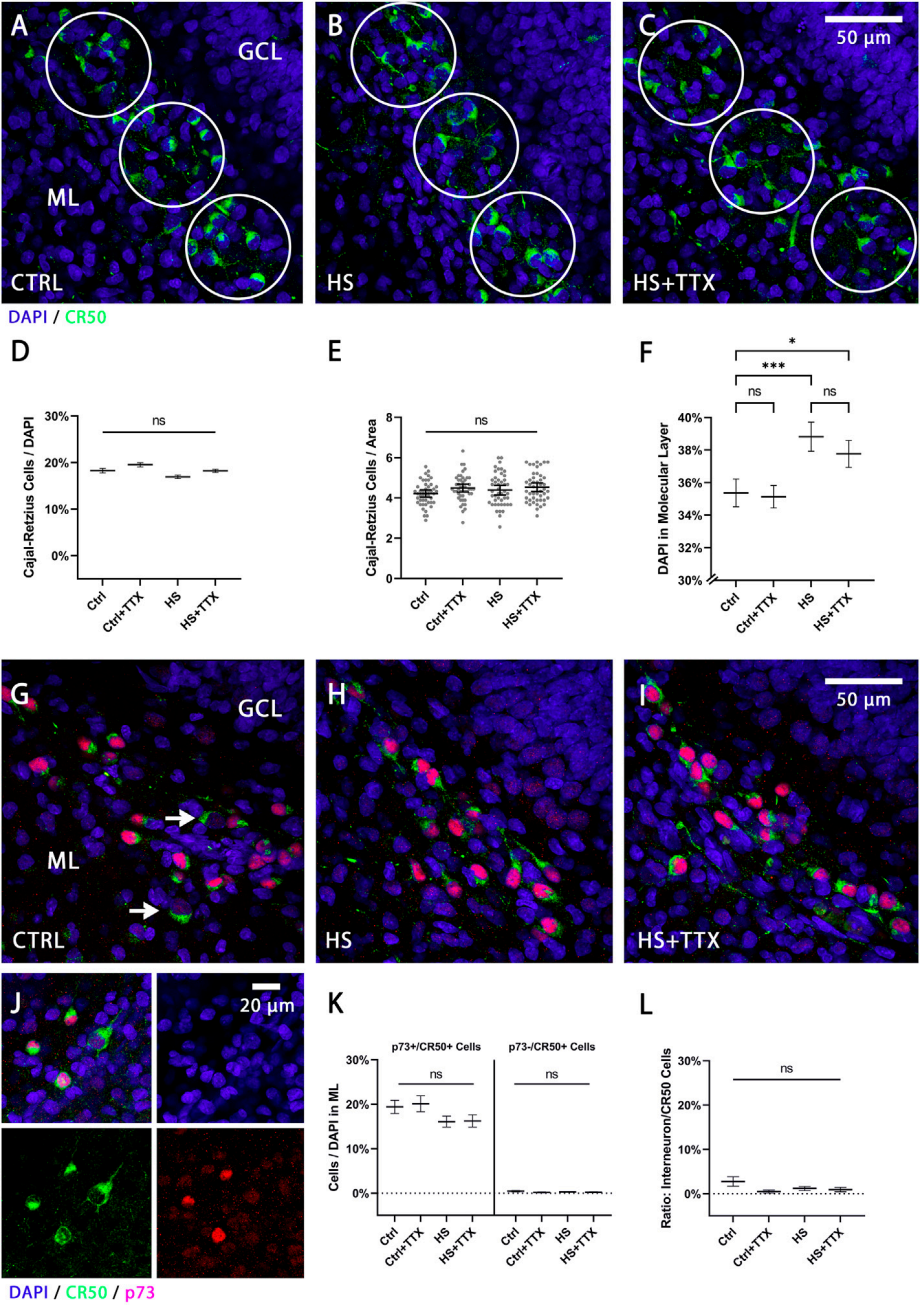
The effect of TTX treatment during heat-shock on Cajal-Retzius cells and reelin-expressing interneurons in the molecular layer. Representative 60x magnification images of animal- and section-matched OHSCs (*n* = 16 animals, 46 OHSCs per condition) for reelin positive Cajal-Retzius cells (CR-50-positive cells in green) in the molecular layer (ML) for **(A)** control (CTRL), **(B)** heat-shock (HS), and **(C)** TTX-treated heat-shock (HS + TTX) OHSCs. Manual cell counts of Cajal-Retzius cells within circular masks was averaged across the ML per slice per condition (from pooled counts in the SB, the CT, and the IB). **(D)** Independent to experimental treatment, there was no significant change in the percentage of Cajal-Retzius cells (ANOVA; Tukey’s post test; mean ± SEM) from the total (DAPI-positive nuclei) number of cells. **(E)** The absolute cell count per unit area (circular masks of 3,500 μm^2^) in the ML was not significantly different across the conditions (ANOVA; Tukey’s post test; mean ± SEM). Data points (grey dots) demonstrate the average count of Cajal-Retzius cells per unit area per slice. **(F)** The percentage of DAPI-positive nuclei (ANOVA; Tukey’s post test; mean ± SEM) in the ML per unit area was significantly increased following HS, with or without TTX treatment, but remained unchanged in TTX-treated control slices. Double staining with p73 and CR-50 (*n* = 4 animals, 12 OHSCs per condition), to distinguish the Cajal-Retzius cells (p73+/CR-50+) from the reelin-expressing interneurons (p73-/CR-50+) in the ML for **(G)** CTRL (white arrows indicating p73-/CR-50+ interneurons), **(H)** HS, and **(I)** HS + TTX OHSCs. **(J)** Split channel view denoting the differential cell size and staining with p73 (red) and CR-50 (green) positive staining for the Cajal-Retzius cells and reelin-expressing interneurons (only CR-50 positive, green). Of note is the increased cell size of interneurons compared to Cajal-Retzius cells. **(K)** Analysis of Cajal-Retzius cells (p73 + /CR−50 + ) and reelin-expressing interneurons (p73−/CR−50+) in the ML indicate the majority of reelin-expressing cells were Cajal-Retzius cells, and differences within the 2 cell groups between the treatment types was not significant. **(L)** The ratio of the p73−/CR-50+ interneurons normalised to CR−50-positive cells indicated the reelin-expressing interneurons account for less than 3% of the CR−50-positive cells in the ML, and any differences between the treatments was not significant. CTRL: control; GCL: granule cell layer; HS: heat-shock; ML: molecular layer; TTX: tetrodotoxin; *p < 0.05, **p < 0.01, ***p < 0.001, and ****p < 0.0001.

The reelin-expressing cell populations, Cajal-Retzius cells and interneurons (distinguishable by size, morphology, and location), were analysed separately (*n* = 16 animals; 46 OHSCs per condition). Manual counts of Cajal-Retzius cells in the molecular layer (Figures 2A–C) were normalised to DAPI-positive nuclei. There was no significant change in the percentage of Cajal-Retzius cells independent to treatment type (Figure 2D). Absolute cell counts per area (circles in Figures 2A–C) in the molecular layer indicated no significant differences in the average cell counts across the treatments (Figure 2E). Whereas TTX treatment in control showed no significant difference in the percentage of DAPI-positive nuclei in the molecular layer (*p* = 0.99; Tukey’s post test; Figure 2F), there was a significant increase in cell number following heat-shock alone (*p* = 0.0006; Tukey’s post test; Figure 2F) as well as with TTX treatment during heat-shock (*p* = 0.03; Tukey’s post test; Figure 2F).

As reelin-expressing interneurons may also account for a population of the CR-50-positive neurons present in the ML, a p73/CR-50 double-staining was performed to confirm previously counted Cajal-Retzius cells (Figures 2G–J). The Cajal-Retzius cells were smaller in cell body size, p73- and CR-50-positively stained (denoted as p73+/CR-50+), while the reelin-expressing interneurons were larger in cell body size, p73-negatively and CR-50-positively stained (denoted as p73-/CR-50+) as observed in Figure 2G (white arrows) and Figure 2J. Analysis confirmed the reelin-positive cells in the molecular layer were almost entirely Cajal-Retzius cells (Figure 2K), and the differences in numbers of Cajal-Retzius cells and interneurons in the molecular layer between the treatments was not significant. At this developmental stage, the p73-/CR-50+ interneurons account for less than 3% of total CR-50-positive cells in the molecular layer (Figure 2L). In all instances, there were no significant differences compared to control in the reelin-expressing cell populations in the molecular layer between treatment types (CTRL + TTX, HS, HS + TTX).

**FIGURE 3:**
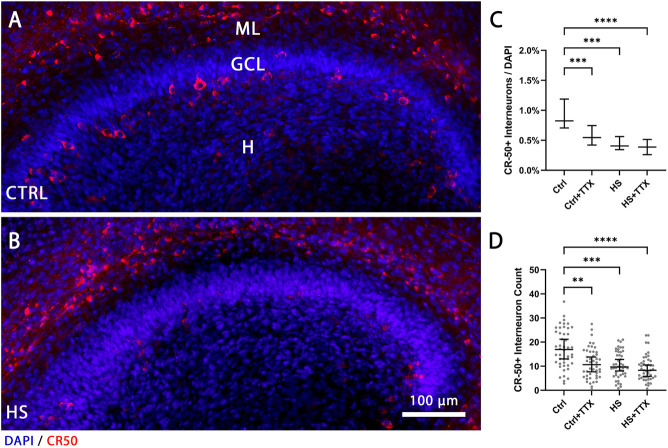
The effect of TTX treatment during heat-shock on the reelin-expressing interneurons in the hilus. Representative 20 × magnification images of animal- and section-matched OHSCs (*n* = 16 animals, 46 OHSCs per condition) for reelin-expressing (CR−50-positive cells in red) interneurons in the hilus (H) demonstrating **(A)** control (CTRL) in comparison to **(B)** heat-shock (HS) interneuron loss. **(C)** Manual cell counts of the cells in the hilus normalised to DAPI and averaged in three z-planes revealed significantly reduced percentage of CR-50-positive interneurons in all experiments compared to control (Friedman test; Dunn’s post test; median ± 95% confidence intervals). **(D)** Absolute cell counts of CR-50-positive interneurons in the dentate gyrus and hilus (grey dots indicating average cell count per slice) demonstrate a similar pattern of CR-50-positive interneuron loss in all experiments compared to control (Friedman test; Dunn’s post test; median ± 95% confidence intervals). CTRL: control; GCL: granule cell layer; ML: molecular layer; (H) hilus; HS: heat-shock; TTX: tetrodotoxin; *p < 0.05, **p < 0.01, ***p < 0.001, and ****p < 0.0001.

Next, we counted CR-50 positive interneurons in the hilus distinguishable by the increased size and morphology (Anstötz and Maccaferri, 2020). The percentage of reelin-expressing interneurons in the hilus before (Figure 3A) and after heat-shock (Figure 3B) was determined by counting CR-50-positive interneurons in the hilus and normalising to the number of DAPI-positive nuclei. There was a significant reduction in CR-50-positive interneurons in the hilus independent to treatment type (*p* < 0.0001; Friedman; Figure 3C). Further, examining the absolute numbers of CR-50-positive interneurons within the dentate gyrus and hilus also indicated a significant reduction (*p* < 0.0001; Friedman; Figure 3D). In both instances, the differences in interneuron loss between treatments (CTRL + TTX, HS, HS + TTX) was not significant.

**FIGURE 4:**
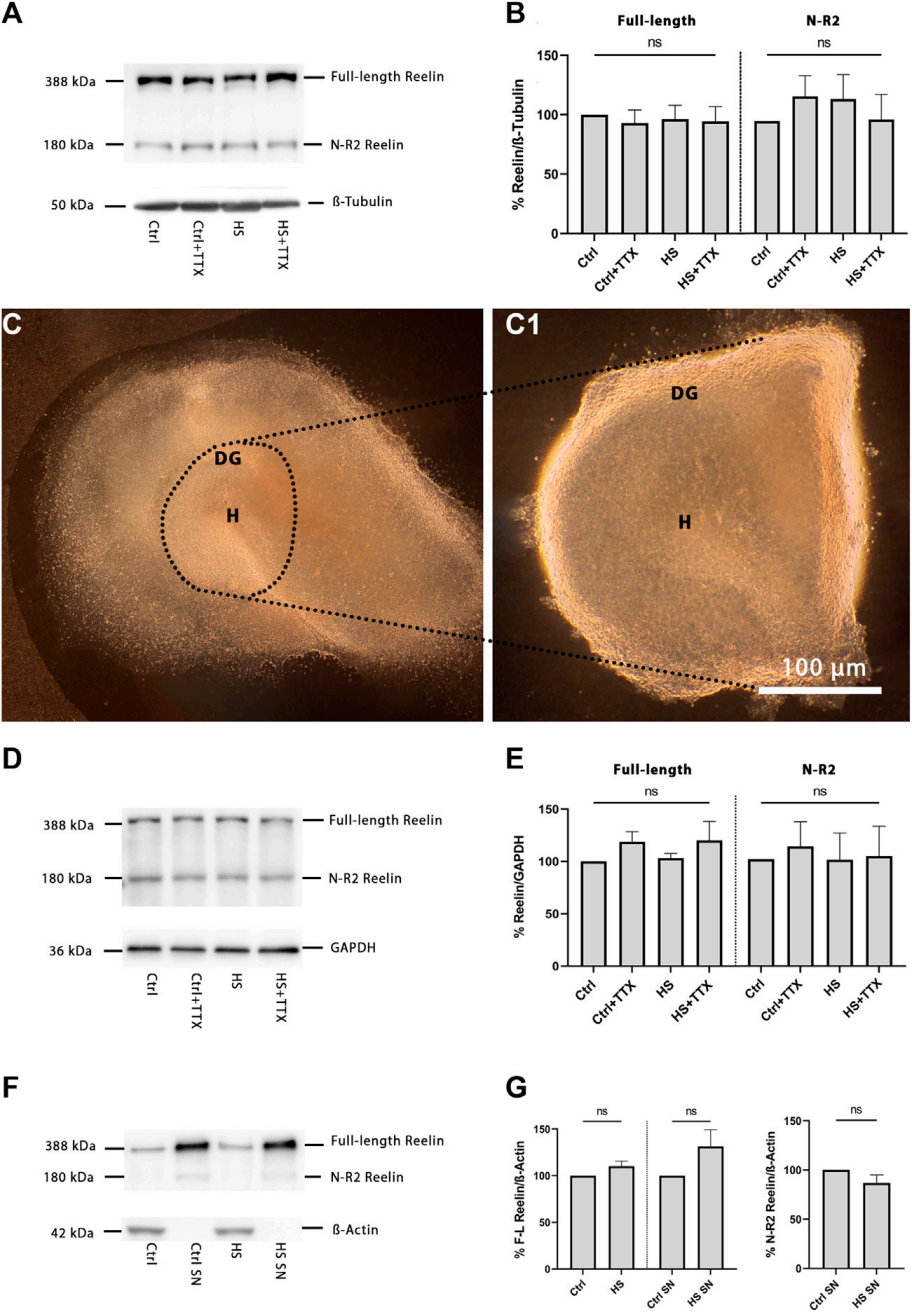
Western blot analysis for reelin. **(A)** Immunoblot of whole OHSCs lysates (*n* = 8 animals, 14 OHSCs per condition) showing bands for 388 kDa full-length reelin and 180 kDa N-R2 reelin. All bands were normalised to the 50 kDa ß-tubulin loading control. **(B)** The protein expression analysis in the TTX experiment yielded no significant differences in either the full-length 388 kDa reelin band, or the 180 kDa reelin band. **(C)** Representative brightfield image of a DIV5 OHSC and **(C1)** after cutout of the dentate gyrus (DG) approximately along the hippocampal fissure including the ML, GCL, and the hilus area from the whole OHSC. **(D)** Immunoblot from DG cutout of OHSCs (*n* = 3 animals, 14 DG cutout of OHSCs per condition) lysates showing bands for 388 kDa full-length reelin and 180 kDa N-R2 reelin. All bands were normalised to the 37 kDa GAPDH loading control. **(E)** Reelin expression analysis from DG cutout of OHSCs was not significantly different irrelevant of the treatment type, for either the full-length 388 kDa reelin band, or the 180 kDa reelin band. **(F)** Immunoblot of full-length reelin transfected HEK293 cell lysates and the corresponding supernatant (SN), indicating bands for full-length reelin at 388 kDa and the N-R2 reelin band at 180 kDa. All bands were normalised to the 42 kDa ß-actin loading control. **(G)** The protein expression analysis in the heat-shock experiment of HEK293 cell lysates yielded no significant differences in either the full-length 388 kDa reelin band for cell lysate or supernatant (SN), or the 180 kDa reelin band in the supernatant (SN) for any of the different treatments. Repeated measures one-way ANOVA; mean ± SEM; TTX: tetrodotoxin; DG: dentate gyrus; GCL: granule cell layer; H: hilus; ML: molecular layer; SN: supernatant; ns: not significant.

To examine changes in reelin expression after heat-shock and TTX treatment, western blot analysis was performed on OHSCs (Figure 4A; *n* = 8). No significant differences in the full-length 388 kDa and the 180 kDa reelin fragment compared to control were observed for the different treatment types (Figure 4B). A more restricted analysis of total reelin expression confined to the dentate gyrus was additionally performed by dissection of the dentate gyrus approximately along the hippocampal fissure including the ML, GCL, and the hilus from the whole OHSC (Figures 4C,C1, *n* = 3), which confirmed the previous results, with no significant differences for the full-length 388 kDa and the 180 kDa reelin fragment (Figures 4D,E). To examine the effect of heat-shock on the expression of the reelin protein and its secretion, a western blot was performed on reelin transfected HEK293 cells (Figure 4F; *n* = 4), where no changes were observed in full-length 388 kDa reelin (in cell lysates or after secretion in the supernatant; Figure 4G) and the 180 kDa reelin fragment (secreted in supernatant; Figure 4G) in heat-shock compared to control.

## Discussion

GCD has been associated as a pathological feature of TLE. Febrile seizures in children have been suggested to increase the propensity to develop TLE (French et al., 1993). In some experimental animal models and in humans with TLE, GCD has been linked with the loss of reelin-expressing cells. We recently introduced an *in vitro* heat-shock model (Weninger et al., 2021) wherein subjecting OHSCs to elevated temperatures induced GCD, but no significant reductions in Cajal-Retzius cells were observed after heat-shock. Here, we attempted to trace the causes that might underlie heat-shock induced GCD. We found that the sodium channel blocker TTX reduced heat-shock induced GCD. Further, no significant change in the percentage of Cajal-Retzius cells in the molecular layer of the dentate gyrus was observed upon TTX treatment after heat-shock, with or without TTX treatment. In contrast, the reelin-expressing interneuron population in the hilus significantly decreased regardless of treatment, whereas quantitative reelin expression in the dentate gyrus did not change significantly. This suggests that heat-shock induced GCD may likely be related to electrical activity rather than to loss of reelin-expressing interneurons.

### Tetrodotoxin prevents heat-shock induced granule cell dispersion

Previously it has been shown that silencing electrical activity of cells with chronic TTX treatment was accompanied by a reduction in cell density (Bausch et al., 2006). It has also been observed that TTX treatment was neuroprotective and reduced the excitotoxic effects of post-insult neuronal network activity cell death (Lahtinen et al., 2001). For instance, in primary hippocampal neuron culture (Jeong et al., 2008), activity blockade with TTX prevented neuronal migration that was observed *via* electrical stimulation. In general, channelopathies may be linked to developmental migrational defects, brain gyrification disorders, and epilepsy (Cappello, 2018). There are a variety of sodium channel subunits linked to intracellular signals that modulate neuronal migration (Schwab et al., 2012). In addition, the sodium channel subunit NaV1.3 is expressed in migratory neurons and radial glial cells, and mutations in this channel are associated with the development of migration disorders and early-life epilepsy (Zaman et al., 2018). Moreover, changes in the activity of the Na-^+^K^+^-2Cl^−^ co-transporter (NKCC1) (Koyama et al., 2012) could be involved in GCD by TTX-manipulated migrational behaviour dysfunction of granule cells at this early developmental stage.

However, since our OHSCs were treated for 8-h with TTX and subsequently cultured for a short period of time (2 days), it may be important to assess morphological changes in a time-dependent manner. In a previous study with OHSCs (Bausch et al., 2006), when field potential recordings were conducted following chronic TTX treatment, spontaneous seizures increased subsequently, however with shorter TTX treatment (24 h) no seizures were observed. As the authors focused on TTX-treatment induced seizures, it is worth examining them in context. Following chronic TTX-treatment, these authors observed a significant increase in action potentials elicited by granule cells, but a decrease in synaptic contacts onto granule cells, indicating that plastic changes in excitatory and inhibitory synaptic contacts were involved. It is likely that time point (before, during, or after heat-shock) and duration (transient or chronic) of activity blockade is crucial in reducing heat-shock induced GCD, and it is important to assess the intricacies of TTX modulation long-term on the granule cells following heat-shock.

In an experimental model of complex febrile seizures (Koyama et al., 2012), granule cell migration changes were observed in an activity-dependent manner. The authors hypothesised that granule cell ectopia alone may not be the cause of epileptiform activity, as spontaneous seizures were not observed in phenobarbital-injected rats despite numerous ectopic granule cells. It may be that granule cell ectopia alone does not cause epilepsy, as it is also observed (albeit to a lesser extent) in the hippocampus of wildtype rats (Scharfman et al., 2003), in non-epileptic paediatric patients (Roy et al., 2020), and it may also develop independent of seizures (Myers et al., 2013). Many migratory granule cells have yet to integrate in the developing dentate gyrus and may be more susceptible to insults or excitation/inhibition imbalance that may halt their layer integration (Hayashi et al., 2015).

Although no change in the number of Cajal-Retzius cells was observed, independent of treatment type, there was a slight increase in the total number of cells in the molecular layer (Figure 2F) due to heat-shock treatment. In a previous study (Weninger et al., 2021), an increase in the number of microglial cells was observed in the molecular layer following heat-shock. Thus, migrating or dividing microglial cells might account for the observed increase in the number of cells in the ML in the present study. In addition, dispersion of granule cells, including newborn granule cells, could contribute this increase. Both increased neurogenesis in the hilus and dentate gyrus (Blümcke et al., 2001; Parent et al., 2005; Haussler et al., 2011; Cho et al., 2015), and depletion of neural stem cells (Sierra et al., 2015) have been associated with epilepsy in humans and in animal models. It has also been shown that OHCS shrink progressively during cultivation *in vitro*, particularly in the z-axis, a process that can lead to an increase in cell number per volume in the GCL over time (Sadgrove et al., 2006). However, in the present study an increased cell number was only observed in the molecular layer in heat-shock treated slice cultures (Figure 2F) when compared to control. Therefore, it is unlikely that tissue shrinkage contributes to the increased cell numbers in the ML, since the number of Cajal-Retzius cells did not change in our heat-shock model. Thus, in the present study, microglial cells, together with dispersed granule cells, are therefore more likely to account for the observed increase in cell numbers.

### Reelin expression is unaffected following heat-shock

Observations of heat-shock-induced GCD point to a reduction in the percentage of reelin-expressing interneurons, but not of Cajal-Retzius cells (Weninger et al., 2021) that produce the majority of reelin at this postnatal stage (Alcántara et al., 1998). Therefore, it appears plausible that reduced reelin expression might cause GCD. Moreover, reelin has been shown to be cleaved by metalloproteinases (Lambert de Rouvroit et al., 1999), and studies have pointed to various functions of the different reelin fragments (Koie et al., 2014; Kohno et al., 2015). In particular, full-length reelin, or more specifically the central reelin fragment, was sufficient to rescue the reeler phenotype *in vitro* (Jossin, 2004). Despite the central-fragment being proposed as the critical factor in rescuing normal lamination, full-length reelin may still be more important due to its ability to self-associate (specifically at the N-terminal region) and form large protein complexes when secreted (Utsunomiya-Tate et al., 2000). However, in the present study, western blot analysis of OHSCs (Figures 4A,B), the isolated dentate gyrus (Figures 4D,E), in reelin-transfected HEK293 cell lysates and secreted full-length reelin in HEK293 supernatant (Figures 4F,G) did not reveal any significant differences in full-length reelin expression, independent to treatment type.

The N-terminal (N-t) Reelin cleavage produces the R3–8 270 kDa and N-R2 180 kDa fragments. In HEK293 cells, after internalisation, reelin was proteolytically cleaved, followed by re-secretion of the N-R2 fragment (Hibi and Hattori, 2009). Thus N-R2 expression has served as an indicator for N-t cleavage (Okugawa et al., 2020). Here, western blot analysis of HEK293 secreted reelin in the supernatant did not reveal any significant differences in N-R2 cleavage following heat-shock (Figures 4F,G). Further, neither in OHSCs nor in isolated dentate gyrus lysates, were any significant differences in N-R2 cleavage observed that might account for GCD (Figures 4A,B,D,E).

Cleavage of reelin has been suggested to be important in releasing the protein anchored to the extracellular matrix (ECM) (Jossin et al., 2007) where it can then exert its effects by binding to its receptors and inducing a signalling cascade. In one study with OHSCs (Tinnes et al., 2010), reelin expression and secretion were unaffected, but its proteolytic processing was hampered concomitant with epileptiform activity (and exhibiting GCD), where reelin accumulation in the ECM was due to inhibition of metalloproteinases. The authors demonstrated an increased expression of full-length reelin in the KA epilepsy model, and decreased levels of the N-R2 fragment in the supernatant. In the present study, western blot analysis (Figures 4F,G) did not indicate a significant difference of N-R2 in the supernatants of HEK293 cells under heat-shock condition compared to control.

Loss of reelin, in particular *via* loss of reelin producing interneurons, was shown to be directly associated with GCD (Haas et al., 2002; Heinrich, 2006; Gong et al., 2007; Duveau et al., 2010), and rescue of GCL lamination was reported by application of recombinant reelin in the KA-induced GCD model (Müller et al., 2009; Orcinha et al., 2016; Orcinha et al., 2021). In contrast, interneuron-specific reelin knockout mice (Pahle et al., 2019) exhibited normal neuronal layering in the dentate gyrus, but the authors also observed an increase in Cajal-Retzius cells, inferring a type of compensatory mechanism involved. Based on our results demonstrating no change in reelin expression after heat-shock and the relatively low numbers of reelin-expressing interneurons in the molecular layer (see Figures 2K,L) a substantial role for proper granule cell layering in the dentate gyrus by reelin-expressing interneurons cannot be assumed.

### Electrical activity as a player in heat-shock induced granule cell dispersion

A TTX effect on Cajal-Retzius cells has been previously shown in cortical explants (Del Río et al., 1996). The disappearance of Cajal-Retzius cells *in vitro* temporally mirrored that of *in vivo* conditions and it was suggested to depend on electrical activity. Therefore, upon chronically treating cortical slice cultures with TTX, the authors observed a six-fold higher number of Cajal-Retzius cells when compared to control, and conversely, co-culturing of OHSCs with entorhinal cortex tissue reduced the number of Cajal-Retzius cells. In the present study, no changes in the number of Cajal-Retzius cells upon TTX treatment were seen (Figures 2D,E,K). In this context, it may be the case that short TTX treatment (8-h, present study) in comparison to chronic TTX treatment (6 days; Del Río et al., 1996) may account for the differences observed. Additionally, reelin expression itself did not appear to be different upon TTX treatment (Figure 4). This was also observed in a study assessing reelin expression following TTX treatment in OHSCs (Tinnes et al., 2010). Therefore, a role of TTX in the survival of Cajal-Retzius cells can be excluded, wherein TTX treatment prevents heat-shock induced GCD. Hippocampal tissue from patients with TLE provided evidence that GCD was not always accompanied by a loss of Cajal-Retzius cells, but that the severity of GCD positively correlated to loss of these reelin-expressing cells (Haas et al., 2002). This might alternatively reflect an increased general loss of cells in TLE, and might not be exclusively due to the loss of Cajal-Retzius cells, as other cell populations are also affected in TLE in both humans (Maglóczky et al., 2000) and animal models (Bouilleret et al., 2000).

In the heat-shock model, the interneuron population was significantly reduced (Figure 3), and this reduction of interneurons was observed regardless of treatment type. Generally, interneurons are particularly susceptible to insults as observed in various animal models of epilepsy (Bouilleret et al., 2000) and in humans (Maglóczky et al., 2000). However, the loss of reelin-expressing interneurons may not necessarily be the cause of the GCD induced by heat-shock. Although there was a significant loss in the reelin-expressing interneurons by treatment with TTX alone, it did not induce GCD. This suggests that an activity-dependent mechanism acting on granule cell migration or granule cell loss may be causally linked to heat-shock induced GCD rather than the loss of reelin-expressing interneurons. What could be the nature of the mechanisms involved? Interestingly, in a pilocarpine-induced epilepsy, 5 days after status epilepticus, there was a significant initial loss of hilar interneurons and GABAergic synapses on granule cells, which rebounded later beyond control levels, suggesting that neurogenesis of granule cells and synaptogenesis of the surviving interneurons may contribute to the pathogenesis (Thind et al., 2009). This is in line with the previously mentioned study of chronic TTX treatment in OHSCs (Bausch et al., 2006), where plasticity of the excitatory and inhibitory synapses onto granule cells was implicated in the propensity of granule cells to express epileptiform activity. Finally, the aforementioned sodium channel subunits may be directly linked to intracellular signals that modulate neuronal migration (Schwab et al., 2012).

On the other hand, Cajal-Retzius cells might have morphological and electrophysiological functions outside of reelin secretion, as observed in reeler mice cross-bred with CXCR4-EGFP (Anstötz et al., 2018), where Cajal-Retzius cells appear to integrate into the circuitry and exhibit GABAergic input and glutamatergic synaptic output. Optogenetic activation of Cajal-Retzius cells points to a glutamatergic output from these cells to stratum lacunosum-moleculare neurogliaform interneurons, suggesting that Cajal-Retzius cells may regulate migration in early developmental stages in an activity-dependent manner (Quattrocolo & Maccaferri, 2014). In addition, ivy/neurogliaform interneurons have also been proposed to mediate activity-dependent regulation of newborn and pre-existing granule cells (Markwardt et al., 2011). In a vGluT2 conditional knockout mouse model (Anstötz et al., 2022), loss of glutamatergic output from Cajal-Retzius cells was implicated in the loss of the feedforward GABAergic input onto granule cells and theorised to result in the increased anxiety observed in behavioural studies.

## Conclusion

Taken together, GCD observed in our heat-shock model was prevented by the sodium channel blocker TTX, suggesting that activity-dependent mechanisms underlie GCD. On the other side, there remain open questions considering the role of reelin in heat-shock induced GCD. It cannot be excluded that transiently elevated temperatures induce more subtle reelin signalling-related effects, such as alterations in the extracellular matrix milieu (Wegrowski, 1993; Grosche and Brückner, 2001; Mizoguchi et al., 2011; Sherman et al., 2015), reelin-related cytoskeletal dynamics important for proper migration of neurons during development (González-Billault et al., 2004; Chai et al., 2009; Meseke et al., 2013), or malformations in the radial glial scaffold concurrent with a particular topographical position of reelin-expressing cells (for reviews: Förster et al., 2010; Förster et al., 2006a, 2006b). Therefore, future studies of the underlying migrational mechanisms in heat-shock induced GCD may yield a better causal understanding of this phenomenon.

## Materials and methods

### Animals

Wistar rats (*n* = 31) from postnatal day 5 or 6 were used for OHSCs, in compliance with regulations set by Ruhr University Bochum and the Animal Welfare Laboratory Animal Regulations (TierSchVerV). Animals were bred and housed in a 12-h light/dark cycle with *ad libitum* food in the animal facility of the medical faculty, RUB.

### Cell culture

HEK293 cells stably transfected with full-length reelin cDNA (D’Arcangelo et al., 1997) were passaged three times and seeded at a rate of 2 × 10^5^/well in a 6-well plate (F-12 K Nut Mix, Gibco; 10% FBS; 1% Penicillin-Streptomycin, Gibco; 0.8 μg/ml G418, ThermoFisher), incubated *in vitro* for 24-h (37°C; 5% CO2) followed by heat-shock (41°C; 5% CO2) for 6-h and after 1 hour recovery (37°C; 5% CO2), the medium was changed. Cells were incubated for two more days, and cell lysate and corresponding supernatant was then used for western blot.

### Organotypic hippocampal slice culture

OHSCs were prepared as previously introduced (Stoppini et al., 1991; Weninger et al., 2021). Four slices (each of 400 µm thickness) side-by-side were selected to create a closely matched set, which was then plated for the four experimental conditions (e.g., control, control + TTX, heat-shock, heat-shock + TTX). From each animal, 2–3 matched sets (8–12 slices) were plated per condition (Merck Millipore Millicell^®^ Cell Culture Inserts; 30 mm PTFE; 0.4 µm) for immunohistochemistry and western blot, and incubated *in vitro* for five days, with medium change every 2 days (900 µl/well; 50% MEM, 25% HBSS, 25% horse serum, 1% 200 mM L-Glutamine, 33 mM glucose, and 0.04% NaHCO₃).

### Heat-shock and pharmacology

Heat-shock was performed as previously described (Weninger et al., 2021). To evaluate the TTX effect, half of the OHSCs under control or heat-shock conditions were subjected to TTX (1 µM) treatment 1 hour before the 6-h heat-shock on DIV3. Heat-shocked OHSCs were allowed to recover for 1 hour at 37°C before medium change. OHSCs then remained at 37°C up to DIV5 (day *in vitro* 5).

### Immunohistochemistry

On DIV5, OHSCs were fixed (4% paraformaldehyde, 0.1 M Phosphate-Buffered Saline, PBS; pH 7.4) for 1 h at room temperature (RT) and washed 3 times with PBS for 20 min. OHSCs were then carefully separated from membrane inserts and immunohistochemistry was performed free-floating on a shaker, sequentially for each antibody. OHSCs were permeabilized with 0.3% Triton X-100 in PBS for 1 hour at RT and incubated with blocking solution (5% Normal Goat Serum, NGS; 2.5% Bovine Serum Albumin, BSA; 0.1% Triton X-100; in PBS) overnight at 4°C. OHSCs were incubated with anti-Reelin CR-50 (D223-3, MBL; 1:1000 in PBS) overnight at 4°C, followed by secondary antibody incubation (Alexa-Fluor 568 or 488 Goat anti-Mouse IgG, Invitrogen; 1:1000 in PBS) for 2 h at RT. They were then incubated with anti-Prox1 (AB5475, Sigma-Aldrich; 1:1000 in PBS) or anti-p73 (AB40658, Abcam; 1:500 in PBS) overnight, 4°C, and incubated with a secondary antibody (Alexa-Fluor 594 or 488 Goat anti-Rabbit IgG, Invitrogen; 1:1000 in PBS). OHSCs were finally stained with DAPI (4′,6-Diamidino-2-phenylindole; 1 μg/ml in PBS, Roche Diagnostics GmbH), washed with PBS and once with dH_2_O, and mounted onto glass slides for analysis. Between all antibody incubation steps, OHSCs were washed with PBS for 20 min, three times.

### Imaging

The immunofluorescent staining of OHSCs was viewed and captured *via* confocal spinning disc microscopy with the VisiView (Visitron Systems GmbH) imaging software. Images were captured at 20 × and 60 × magnification.

### Western blot

On DIV5, whole OHSCs were picked from the membrane insert with a spatula, quickly submerged in lysis buffer (80 µl/sample; 100 mM Tris-HCl, pH 7.4; 12 mM Magnesium acetate tetrahydrate; 6 M Urea; PhosSTOP Phosphatase Inhibitor, cOmplete™ ULTRA Protease Inhibitor, Roche Diagnostics GmbH) and flash-frozen. For quantitative analysis of reelin in the dentate gyrus, the dentate gyrus was cut out from OHSCs with a scalpel, lysed (35 µl lysis buffer/sample) and flash frozen. HEK293 cells were harvested using a cell scraper and lysis buffer (200 µl/well of a 6-well plate) and flash-frozen. Approximately 30 µg of protein was loaded onto 10% SDS-PAGE gels, gels were transferred to a nitrocellulose membrane and washed in PBS for 5 min. Membranes were blocked with 5% BSA in PBS for 1 h at RT and incubated with the anti-Reelin G10 antibody (MAB5364, Sigma-Aldrich; 1:500, 5% BSA in PBS) and the loading control antibody (for OHSCs: anti-ß-tubulin T4026, Sigma-Aldrich; or anti-GAPDH G8795, Sigma-Aldrich; for HEK293 cells: anti-ß-actin, A5441, Sigma-Aldrich; 1:10,000 in 5% BSA in PBS) overnight at 4°C on a shaker and washed in 0.1% Tween-20 in PBS three times, 20 min each. Secondary antibody was added for 1 h (anti-Mouse HRP-conjugated secondary antibody, Thermofisher; 1:10,000 in PBS) and washed as previously described. Membranes were incubated for 5 min with developing solution (Vilber PURECL^TM^ Ultra Substrate) for chemiluminescent detection and visualised with Vilber Fusion FX imaging. Quantification of protein bands was performed *via* densitometric analysis with ß-tubulin or GAPDH as the loading control for OHSCs and ß-actin for HEK293 cells using the ImageJ gel analysis tool. Ratios were normalised to internal control for each dataset (control was set to 100%), to produce a percentage value for the change in protein expression compared to control.

### Cell counting

Average density of Prox1-positive cells was normalised to DAPI-positive nuclei, space between Prox1-positive nuclei was measured in 60 × resolution in an automated threshold-dependent way *via* ImageJ as previously described (Weninger et al., 2021). Briefly, in each dentate gyrus section, on suprapyramidal blade (SB), crest (CT), and infrapyramidal blade (IB), three circular masks (−2,000 μm^2^; Figures 1A1–C1) were positioned for sampling the cell density and compactness of Prox1-positive nuclei of granule cells. In 60 × magnification, three circular masks (−3,500 μm^2^; Figures 2A–C) per section, spanning the molecular layer, were used for sampling the average cell density of Cajal-Retzius cells by manually counting the CR-50-positive (and p73+/CR-50+) Cajal-Retzius cells which were normalised to DAPI-positive nuclei. Similarly, reelin-expressing interneurons (p73-/CR-50+) in the molecular layer were manually counted and normalised to DAPI-positive nuclei. The CR-50-positive interneurons were manually counted within the hilus and normalised to DAPI-positive nuclei. The hilus area was defined by using Prox1-and DAPI-positive images of the dentate gyrus. A straight line was drawn as an extension of the CA3 pyramidal cell layer, crossing the hilus and the dentate gyrus crest region. Next, a line was drawn in a right angle to it from the end of the suprapyramidal to the infrapyramidal blade (dashed line; Figures 1G–I), thereby defining a standardised region of interest in the hilus. The region of interest was used to analyse whether the hilus size changed across the different experimental conditions.

### Granule cell layer width and area measurement

Width measurements of GCL were taken in triplicates in 60 × magnification for each dentate gyrus section (SB, CT, IB; Figures 1A–C) as previously described with minor changes (Weninger et al., 2021) and averaged per slice. Briefly, three measurements (−80 µm distance apart) for each dentate gyrus section were taken by measuring the shortest distance from the hilar border of the GCL to the outer border of the most distal granule cell somata, while Prox1-positive nuclei in a distance of more than 30 µm from the GCL were excluded from the measurement. To further analyse GCL morphology, a mask area was drawn around the dentate gyrus to assess the total area per slice (dashed lines; Figures 1G–I).

### Statistical analysis.

All data was collected and analysed using Microsoft Excel, Minitab 19, and GraphPad Prism 9. Data were checked for homoscedasticity with Levene’s test and normality with D’Agostino-Pearson or Anderson-Darling test. For normally distributed data, repeated-measures one-way ANOVA was used, significant differences were assessed with Tukey’s post hoc test. For non-normal data, Friedman’s test was performed, followed by Dunn’s post hoc test. Data are presented as mean ± SEM (standard error of mean) for normally distributed data. Data are presented as median ±95% CI (confidence intervals) or boxplots with median, 25% and 75% quartiles for non-normal data. Significance was designated as *p < 0.05, **p < 0.01, ***p < 0.001, and ****p < 0.0001.

## Data Availability

The raw data supporting the conclusion of this article will be made available by the authors, without undue reservation.
